# Enhanced Abiotic Stress Tolerance of *Vicia faba* L. Plants Heterologously Expressing the *PR10a* Gene from Potato

**DOI:** 10.3390/plants10010173

**Published:** 2021-01-18

**Authors:** Abeer F. Desouky, Ahmed H. Hanafy Ahmed, Hartmut Stützel, Hans-Jörg Jacobsen, Yi-Chen Pao, Moemen S. Hanafy

**Affiliations:** 1 Plant Biotechnology Department, Genetic Engineering and Biotechnology Division, National Research Centre (NRC), Tahrir Str. Dokki, 12311 Cairo, Egypt; abeerbiotech@yahoo.com; 2 Agricultural Botany Department, Plant Physiology Section, Faculty of Agriculture, Cairo University, 12613 Giza, Egypt; drahmedhanafy@yahoo.com; 3 Institute of Horticultural Production Systems, Leibniz Universität Hannover, Herrenhäuser Straße 2, 30419 Hannover, Germany; stuetzel@gem.uni-hannover.de (H.S.); pao@gem.uni-hannover.de (Y.-C.P.); 4 Institute of Plant Genetics, Section of Plant Biotechnology, Leibniz Universität Hannover, Herrenhäuser Str. 2, 30419 Hannover, Germany; hj_jacobsen@mac.com

**Keywords:** abiotic stress, gas exchange, pathogenesis-related (PR) proteins, transgenic plant, *Vicia faba* L.

## Abstract

Pathogenesis-related (PR) proteins are known to play relevant roles in plant defense against biotic and abiotic stresses. In the present study, we characterize the response of transgenic faba bean (*Vicia faba* L.) plants encoding a *PR10a* gene from potato (*Solanum tuberosum* L.) to salinity and drought. The transgene was under the mannopine synthetase (pMAS) promoter. *PR10a*-overexpressing faba bean plants showed better growth than the wild-type plants after 14 days of drought stress and 30 days of salt stress under hydroponic growth conditions. After removing the stress, the PR10a-plants returned to a normal state, while the wild-type plants could not be restored. Most importantly, there was no phenotypic difference between transgenic and non-transgenic faba bean plants under well-watered conditions. Evaluation of physiological parameters during salt stress showed lower Na^+^-content in the leaves of the transgenic plants, which would reduce the toxic effect. In addition, PR10a-plants were able to maintain vegetative growth and experienced fewer photosystem changes under both stresses and a lower level of osmotic stress injury under salt stress compared to wild-type plants. Taken together, our findings suggest that the *PR10a* gene from potato plays an important role in abiotic stress tolerance, probably by activation of stress-related physiological processes.

## 1. Introduction

Drought and salinity are the most prevalent abiotic causes of reduced plant growth and productivity worldwide [1,2]. With climate change on the way, the problems will increase dramatically. Therefore, research to enhance the resilience of crops to combat climate change by using modern breeding tools is mandatory. As some plant species or genera cope with these challenges by developing several adaptive features at physiological, biochemical, molecular, and morphological levels, such as accumulating proteins, osmoprotectants, regulating ion absorption, scavenging reactive oxygen, and water balance, involving the function(s) of single or multiple genes [3,4,5]. All these mechanisms can be employed to acquire desirable traits in plant breeding programs. Drought and salt stresses have negative effects on various physiological processes, in particular photosynthesis, which represents the main cause of growth suppression [6,7,8]. As an example, under severe water deficit, one of the first responses of plants often is decreased stomatal conductance to avoid low water potentials, followed by changes in root architecture to maximize water uptake [9]. As a consequence, photosynthesis is reduced due to the stomatal limitation of CO_2_ uptake [10]. The outcome is a loss of plant growth and productivity, and the plant may die under prolonged water stress.

Complex traits, such as abiotic stress tolerances, are difficult to select for using classical breeding. Therefore, one promising approach is to integrate drought/salt tolerance traits through genetic engineering into existing varieties to improve drought/salt tolerance. Genes carrying these traits are now coming on line and, applied together with traditional breeding, may offer the farmers new and sustainable varieties capable of responding to conditions of limited water availability. The engineering of drought/salt-tolerant crop plants has been a long-held and intensively sought objective. A wide range of genes encoding different structural and regulatory proteins have been reported and/or have been employed over the past decades to develop abiotic stress-tolerant transgenic plants [11].

Pathogenesis-related (PR) proteins accumulate in plants upon pathogen infestations and, in many cases, in response to abiotic stresses for survival [12,13,14,15,16]. The major families of PR proteins have been grouped into 17 different classes, primarily based on their structural and functional properties [17,18]. Among them, PR10 is the largest family of all classes of PR proteins, which have various antimicrobial and ribonuclease activities. In addition, PR10 proteins are well known as osmotically inducible proteins and elicitors [19]. Therefore, PR10 proteins play an important role in plant defense responses against biotic and abiotic stresses [20,21,22,23,24,25,26,27,28,29]. The role of PR10 proteins in meditating responses to salinity has been studied by proteomic investigation of pea under salt stress. These investigations revealed a significant increase in the level of several classes of PR 10 proteins, which led to hypothesizing the importance of PR10 proteins in meditating salt stress [30]. Overexpression of PR10 (ABR17) proteins caused enhanced abiotic stress tolerance in both *Brassica napus* and *Arabidopsis thaliana* [31,32]. Moreover, proteome analysis of rice roots under salinity and drought stresses also demonstrated the induction of PR10 protein expression [33]. Heterologous overexpression of PR10 protein from *Panax ginseng* in *Arabidopsis thaliana* enhanced salt stress tolerance with increased root length [34]. The group of PR10 proteins was defined by [17]. This group includes the PR10a protein from potato [35].

Despite the efforts of several research groups and the published studies, the precise function of many PR proteins is not fully known [28]. In the literature, many studies about the involvement of PR10 proteins in plant tolerance to abiotic stress can be found. For example, rice *RSOsPR10* and *Osdrr* (encoding a PR10 protein) genes were found to be up-regulated in rice roots when subjected to drought and salt stress [33,36]. Moreover, PR10 protein levels were highly induced in rice seedlings and leaves under high ozone [37] and UV-mediated stresses [38]. The constitutive expression of a pea *PR10* gene in *Brassica napus* enhanced germination and growth rates under salinity [12]. Proteomic studies revealed that PR10 proteins were accumulated in response to salt stress in grass pea, in a salt-tolerant barley variety, and in the wine grape cultivar Cabernet Sauvignon [39,40,41]. The comparison between a salinity tolerant peanut callus cell line against its sensitive counterpart revealed that most of the differently abundant low molecular proteins were PR10 [22]. Moreover, *Arabidopsis thaliana* seedlings overexpressing *SmPR10* from *Salix matsudana* Koidz had a higher Na^+^ uptake capacity in the roots and higher salt tolerance compared to WT plants [42]. Thus, the manipulation of PR10 protein abundance was shown as a particularly promising approach to improve abiotic stress tolerance in crops [28]. 

Faba bean (*Vicia faba* L.), an important pulse crop, is grown as staple food and animal feed worldwide [43]. In addition to the seeds of faba bean being a rich source of proteins, carbohydrates, fiber, and minerals, the crop improves soil fertility via fixing atmospheric nitrogen (N_2_) in association with rhizobia. However, soil salinity and drought represent major constraints to the productivity of faba beans. In an earlier study on proteome analysis of potato cell culture subjected to salt and osmotic challenges, it was shown that the PR10a protein was upregulated [44]. In our previous study, the *PR10a* gene from potato was overexpressed in faba bean plants after *Agrobacterium*-mediated transformation with the aim of obtaining faba bean with improved tolerance to drought and salt stresses [15]. In the present study, in addition to phenotype observation and ion analysis, the physiological performance of the transgenic plants under salt and drought stresses was evaluated by analyzing gas exchange parameters, relative chlorophyll content (SPAD value in fresh leaves), and leaf osmotic potential. Since these parameters reflect the integrity of photosystem II and stomatal responses upon exposure to water stress, these analyses provide information on salt and drought responses of the transgenic faba bean plants.

## 2. Results

### 2.1. Screening of PR10a Transgenic Plants

**Figure 1:**
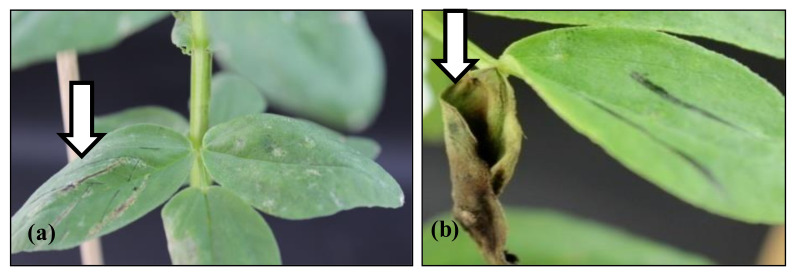
Herbicide leaf paint test showing (**a**) resistance of a transgenic plant to BASTA^®^ application (300 mg/L ammonium glufosinate); (**b**) the control non-transgenic leaf dying after BASTA^®^ application.

**Figure 2:**
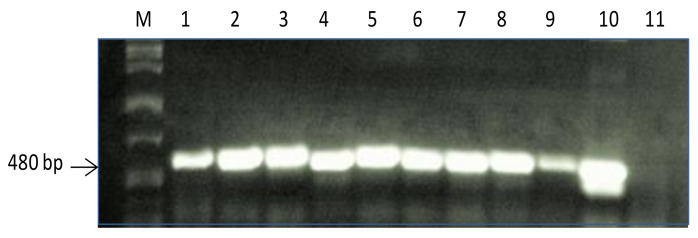
PCR analysis of PR10a faba bean: *lane M* DNA Molecular weight marker, *lanes 1–9* transgenic faba bean plants, *lane 10* plasmid DNA, *lane 11* non-transformed control plants. The size of the amplified fragment is 480 bp.

First, stable transgenic faba bean plants containing the *PR10a* gene were screened by a leaf paint test and PCR analysis using specific primers for the *PR10a* gene (Figure 1 and Figure 2) to select positively expressing plants from the segregating ones. After the paint test, transgenic plants showed complete resistance to herbicide application and showed the band of the expected size of *PR10a* gene (480 bp). Generally, the segregation ratios did not significantly differ from the expected 3:1 segregation ratio for the inheritance of a single transgenic locus after self-pollination, or a completely transformed offspring (progeny of line TL-3).

### 2.2. Growth Reactions of PR10a Transgenic Faba Bean Lines to Salt Stress

**Figure 3:**
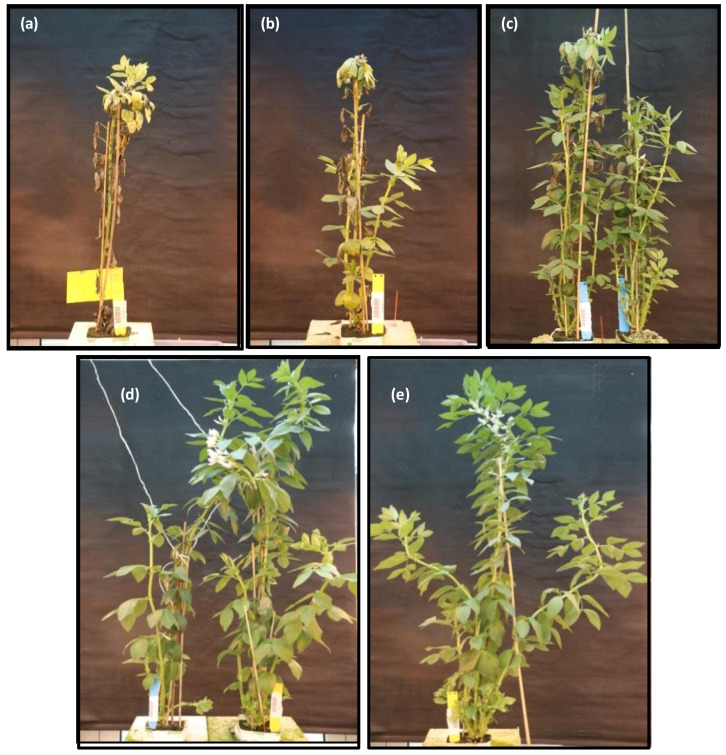
Phenotypes of wild-type (**a**,**e**) and PR10a faba bean lines TL-2 (**b**), and TL-3 (**c**,**d**) with (**a**–**c**) and without (**d**–**e**) salt stress for 29 days followed by 14 days without salt stress (**a**).

To assess whether the heterologous expression of *PR10a* in faba bean is associated with salt stress tolerance, two transgenic lines; TL-2 (low expression level of *PR10a*) and TL-3 (high expression level of *PR10a*) [15], along with wild-type (WT) plants were subjected to salt stress by the gradual increase in NaCl levels (50, 100, and 150 mM NaCl) for 25 days in a hydroponic culture system. No morphological differences were observed between WT and PR10a plants in the treatment without salt stress (Figure 3d,e). The difference in salt tolerance between wild-type and PR10a plants was obvious with increasing the NaCl concentration and the period of exposure to salt stress. Distinct differences were observed in the *PR10a*-overexpressing faba bean and WT plants at the end of the salt treatment (Figure 3a–c). Wild-type plants displayed progressive chlorosis and general growth inhibition with the gradual rise in NaCl concentration. Wild-type plants grown in the salt treatment displayed a severe reduction in size. Plants of the PR10a line TL-3 grew better than the WT in the salt treatment, but those of faba line TL-2 showed more chlorosis (Figure 3). These results suggested that the overexpression of the *PR10a* gene in faba bean plants promotes sustained growth and development under salt stress. 

**Figure 4:**
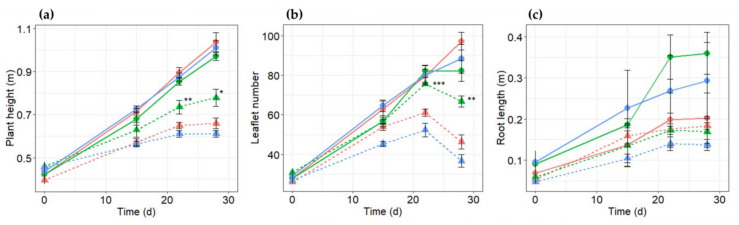
Changes in (**a**) plant height, (**b**) leaflet number per plant, and (**c**) root length in faba bean plants; PR10a transgenic lines TL-3 (green), TL-2 (red), and wild-type faba bean (blue) under non-stressed (circular symbols and full lines) and salt stress conditions (triangle symbols and dashed lines). Data points represent the means (±SE) of at least three replicates.

To further study the difference between the transgenic and WT plants during salt treatment, plant height, leaf number per plant, root length, branches number per plant, and plant biomass were measured after exposure to salt stress (Figure 4, Figure S1, and Table 1 and Table 2). No obvious phenotype differences were observed between the WT and transgenic plants before salt treatments. Although these parameters decreased gradually with an increased salt concentration in all plants, plant height, number of leaflets, and branch per plant were significantly lower in the WT plants than in the transgenic plants, particularly in the transgenic line TL-3 (*p* < 0.05).

The growth parameters were measured on days 15, 22, and 28 after the beginning of salt stress treatment. Average plant height and number of leaflets per plant of line TL-3 treated with NaCl were significantly higher than WT and TL-2 plants (Figure 4a,b), while no significant difference was found between lines in root length (Figure 4c). Interestingly, although the reduction in plant height and leaflet number was significantly higher in transgenic line TL-2 under salt stress compared to transgenic line TL-3, the reduction in root length of transgenic line TL-2 was only 7.1% under salt stress compared to the corresponding plants growing under normal condition. Therefore, another study should be carried out to explain these interesting phenomena. 

After growing for 29 days under salt stress followed by 14 days without salt stress, there were no significant differences in total, root, and leaf fresh weight between the transgenic lines and WT (Table 1 and Figure S1a–c). However, looking at relative values, TL-3 had significantly higher fresh weights of all organs than WT after stress. TL-3 also had a significantly higher number of branches per plant compared to TL-2 and WT. Insignificant line x treatment interactions showed that total plant dry weight, leaf, stem, and root dry weights of PR10a lines and WT plants were reduced similarly by salt stress (Table 2). However, relative total, leaf and stem dry weights of TL-3 were significantly higher than of WT. The reductions in dry weights of total plant dry weights, stem dry weight, leaf dry weight, and roots dry weights were less severe in transgenic lines with no significant differences, compared to those observed in the WT plants under salt stress condition (Table 2).

### 2.3. Effects of Salt Stress on Gas Exchange Parameters in PR10a Faba Bean Lines

**Figure 5:**
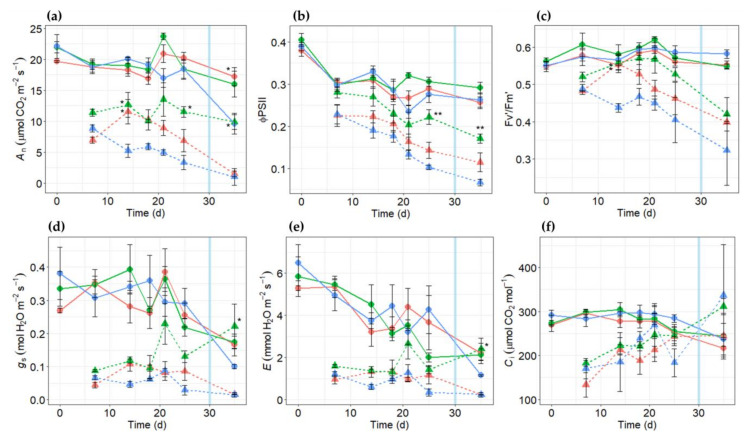
Gas exchange in faba bean PR10a transgenic lines; TL-3 (green), TL-2 (red), and wild-type (blue) under non-stressed (circular symbols and solid lines) and under salt stress conditions (triangle symbols and dashed lines) on days 7, 14, 18, 21, and 25 from the salt application. The salt was removed on day 30 (light blue line). (**a**) Net photosynthetic rate *A*_n_, (**b**) quantum efficiency of photosystem II electron transport under 1200 µmol m^−2^ s^−1^ PAR (ϕPSII), (**c**) maximum efficiency of photosystem II photochemistry in the light Fv’/Fm’, (**d**) stomatal conductance *g*_s,_ (**e**) transpiration rate *E* and (**f**) intercellular CO_2_ concentration *C*_i_. Data points represent means (±SE) of at least four replicates.

To evaluate salt tolerance in the vegetative stage, PR10a and wild-type (WT) plants were grown hydroponically under salt stress in a greenhouse. In general, net photosynthetic rate (*A*_n_), quantum efficiency of photosystem II electron transport (ϕPSII), and maximum efficiency of photosystem II photochemistry (Fv’/Fm’) measured under 1200 µmol m^−2^ s^−1^ PAR, stomatal conductance, transpiration rate, and intercellular CO_2_ concentration decreased rapidly in all plants upon salt stress treatment (Figure 5, day 7, significant treatment effect with *p* < 0.05). The gradual decrease along exposure to salt stress was more obvious in *A*_n_, ϕPSII, and Fv’/Fm’ (Figure 5a–c). However, stomatal conductance and transpiration rate stayed at about the same levels after day 7 (Figure 5d,e), and intercellular CO_2_ increased with time under salt stress (Figure 5f). Parameters *A*_n_ and ϕPSII were significantly lower in WT plants as compared to TL-3 at day 25 after the onset of salt stress treatment. Moreover, TL-3 showed significantly higher levels of gas-exchange attributes than TL-2, especially under severe salt stress (at day 25 after salt treatment; Figure 5), indicating that the PR10a plants exhibited greater tolerance to salt stress. 

While PR10a line TL-3 maintained higher net photosynthetic rates (*A*_n_) than WT under salt stress (Figure 5a), PR10a line TL-2 showed lower *A*_n_ than WT at the beginning of salt stress with 100 mM NaCl. Afterward, TL-2 recovered and remained at higher *A*_n_ than WT. PR10a line TL-3 exhibited significantly higher *A*_n_ as compared to WT on days 14 and 25 after the onset of salt stress. After 25 days of salt treatment, *A*_n_ of WT was reduced by about 82%, while the reduction in TL-3 was only 37.1% (Figure 5a). It might be inferred that overexpression of *PR10a* in faba bean could improve the efficiency of photosynthetic performance.

A similar tendency was observed for ϕPSII, Fv’/Fm’, stomatal conductance (*g*_s_), and transpiration rate (*E*), with the WT plants being impaired more by salt stress than the transgenic lines (Figure 5). The reduction in leaf intercellular CO_2_ concentration (*C*_i_) induced by NaCl application was less in PR10a than in WT plants on day 25 after the onset of salt application (Figure 5f). The values of *E* were correlated with the obtained results of *g*_s_. WT plants showed the highest reduction in *g*_s_ and *E* (Figure 5) at the end of the salt stress period. After removing the salt stress, the TL-3 recovered and showed wider opened stomata than the plants growing under non-stress conditions. Observed *g*_s_ and *E* were higher by 19% and 12.2%, respectively, at the recovery stage (after 5 days after removing the salt stress) compared with the plants grown under non-stressed conditions. Interestingly, PR10a line TL-2 and WT plants failed to recover after removing the salt stress. 

### 2.4. Osmotic Potential

Leaf osmotic potentials were measured using psychrometer cells 14 and 28 days after salt stress application. Osmotic potentials of the leaves were similar for WT and PR10a lines under non-stressed conditions but were significantly different after salt stress (Table 3). Leaf osmotic potential decreased significantly in both PR10a lines and WT plants after exposure to salt stress. A non-significant difference in osmotic potential was observed in PR10a line TL-3 and WT plants after two weeks of salt stress application (Table 3). A gradual decrease in leaf osmotic potentials with increasing stress duration was observed (Data not shown). The maximum reduction in leaf osmotic potential due to salt stress was observed after 28 days of salt stress application. The osmotic potentials declined from about −0.74 MPa (non-stressed) to −1.91 MPa in the leaves of WT plants after 28 days of salt stress, whereas PR10a lines TL-2 and TL-3 maintained their leaf osmotic potentials at about −1.3 MPa (Table 3).

### 2.5. Chlorophyll Content Estimation by SPAD Chlorophyll Meter

**Figure 6:**
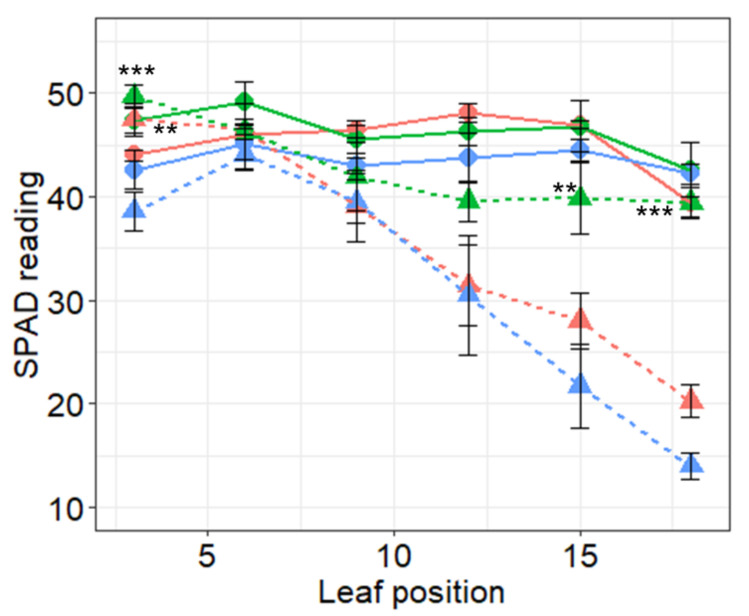
Chlorophyll index (SPAD) readings of faba bean PR10a transgenic lines TL-3 (green), TL-2 (red), and wild-type (blue) under non-stressed (circular symbols and solid lines) and salt stress conditions (triangle symbols and dashed lines) after 25 days of salt stress application. The measurement was carried out on leaves no. 3, 6, 12, 15, and 18 (from base to top), with three readings repeated for four sample plants. Data points represent the means ± SE. Asterisks indicate significant differences within treatment by Dunnett’s test using WT as control upon significant interaction indicated by two-way ANOVA. *** *p* < 0.001, ** *p* < 0.01, n, insignificance with *p* > 0.05.

The soil plant analysis development (SPAD index) is widely used to evaluate changes in chlorophyll content. SPAD readings were taken from fully expanded leaves at nodal positions 3, 6, 9, 12, 15, and 18 counted from the base to the top of plants after 25 days of salt treatment. Although decreasing SPAD values were observed in both transgenic and WT plants under salt stress, the decrease in WT was significantly higher compared to PR10a line TL-3 at nodal positions 3, 15, and 18 (Figure 6). 

### 2.6. Ion Analysis

Following salt stress for 29 days, a 14-day recovery was conducted. After the recovery stage, sodium (Na^+^) and chloride (Cl^−^) ions were analyzed. As expected, salt stress resulted in a sharp increase in leaf Na^+^ and Cl^−^ contents (Table 4 and Figure S2), with WT plants showing significantly higher accumulation of Na^+^ in leaves than PR10a lines TL-2 and TL-3. No significant effect of either line or treatment was observed in the accumulation of Na^+^ in the stems (Table 4). On the other hand, salt stress markedly increased the root Na^+^ content in PR10a line TL-3 when compared with WT (Table 4). Although Cl^−^ concentrations sharply increased in plant tissues under salt stress, there was no significant interaction between line and treatment (Figure S2). 

Under salinity, both PR10a and WT plants exhibited significantly lower leaf potassium (K^+^), calcium (Ca^2+^), and magnesium (Mg^2+^) contents than in the controls with no significant differences between lines (Figures S3a, S4a, and S5a). Similarly, stem and root K^+^ contents tended to be lower under salt stress (Figure S3b,c), while root Ca^2+^ (Figure S4c) and stem Mg^2+^ (Figure S5b) contents tended to increase, with insignificant differences between transgenic lines and WT. 

### 2.7. Phenotyping of the PR10a Faba Bean Plants under Drought Stress

**Figure 7:**
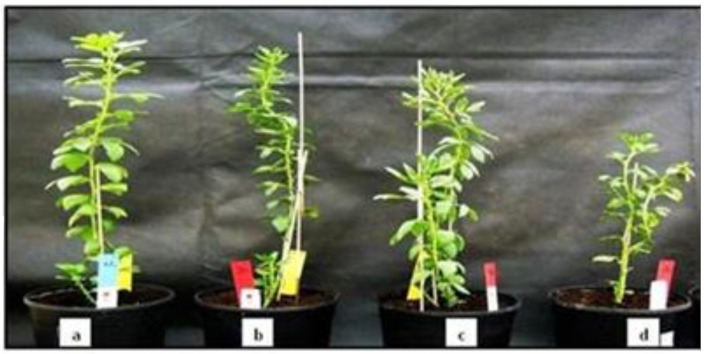
Plant growth under non-stressed and drought-stressed conditions. (**a**) Growth of PR10a transgenic plant under non-stressed condition, (**b**,**c**) PR10a transgenic plants under drought stress for two weeks, (**d**) wild-type under drought stress for two weeks.

To investigate whether overexpression of *PR10a* improved drought stress tolerance, different generations of faba bean PR10a transgenic line TL-2 was subjected to a water deficit regime for 14 days, followed by a 2-day recovery period. No obvious morphological and developmental differences were observed between transgenic and WT plants under non-stressed conditions (data not shown), except for that TL-2.2 and TL-2.3 showed higher plant height and more leaves per plant (Table 5). Leaves of WT plants showed severe wilting symptoms, whereas transgenic plants showed lesser wilting signs after two weeks of drought stress (Figure 7). Drought stress had adverse effects on the growth of WT plants (Figure 7d), such that plant height and leaf number per plant were significantly reduced in WT, while transgenic lines could maintain their plant heights after two weeks of drought stress (Table 5). After rewatering, transgenic plants recovered better and more quickly, whereas the WT plants failed to recover (data not shown). These observations suggest heterologous overexpression of the *PR10a* gene conferred increased drought tolerance in faba bean plants. 

### 2.8. Measurement of Physiological Changes under Drought

Net photosynthetic rate (*A*_n_), quantum efficiency of photosystem II electron transport in light (ϕPSII), stomatal conductance (*g*_s_)_,_ transpiration rate (*E*), and intercellular CO_2_ concentration (*C*_i_) were determined to evaluate the drought tolerance of *PR10a*-overexpressing faba bean plants. Under the non-stressed condition, there was no significant difference in all measured parameters between WT and transgenic plants (Table 6). After 14 days of drought stress, *g*_s_ significantly decreased in all plants, indicating a general stress response of stomatal closure, which led to a reduction in *E*. Upon closing stomata under drought stress, CO_2_ influx into leaves also decreased. Thus, lower *C*_i_ of all transgenic lines TL-2.2, TL-2.3, and TL-2.4 under the stressed condition compared to their counterparts under the non-stressed condition and compared to WT under stress suggests a better maintenance of carboxylation functioning in transgenic plants. In addition, only the WT showed a significant reduction in ϕPSII under stress, indicating that WT plants were not able to maintain photosystem II electron transport as transgenic lines. However, only transgenic line TL-2.3 showed significantly higher *A*_n_ under stress than WT, which highlights the role of carboxylation in photosynthetic performance under drought stress. Two days after rewatering, *g*_s_ and *E* were able to recover to those as under non-stressed condition in all plants, while WT plants failed to recover in *A*_n_ and ϕPSII (Table 6).

## 3. Discussion

Drought and salinity are abiotic environmental stresses threatening modern agricultural productivity worldwide [45,46]. Abiotic stress exerts its negative impacts on most plant processes, such as disrupting the ionic and osmotic equilibrium, photosynthesis, or protein synthesis. Thus, abiotic stress causes adverse effects on plant growth and biomass [6,47,48]. Plant response to abiotic stress involves alterations of various physiological and biochemical processes dependent upon the crop and time of exposure [49,50]. Salinity and drought stresses cause oxidative stress and a reduction in photosynthetic capacity [48]. Photosynthesis reduction results from a reduction in CO_2_ diffusion into plant leaves due to lower internal (*g*_i_) and stomatal conductance (*g*_s_). The inhibition of photosynthesis is also due to limited cell proliferation and leaf growth under stress [51,52,53]. Chlorophyll fluorescence is a vital indicator of different salt and drought responses of photosynthesis [54]. The measurements of gas-exchange parameters in the intact, attached leaves were shown to be useful, non-invasive, and reliable for monitoring photosynthetic events and studying the physiological status of the plant [55,56,57].

In the present study, the growth of *PR10a*-overexpressing faba bean plants was obviously better than that of wild-type plants under drought and salinity conditions. Importantly, transgenic PR10a faba bean plants showed no obvious morphological differences from the wild-type plants under normal conditions.

The enhanced salinity tolerance was measured by several parameters, such as plant height, leaf number per plant, root length, and quantifying plant total fresh/dry weights and its partitions. Transgenic line TL-3 showed improved tolerance to salt stress in terms of maintaining plant height, number of leaves per plant, number of branches per plant as well as relative dry and fresh weights better than wild-type plants (Table 1). The fact that absolute dry weights did not show significant improvements in TL-3 may be due to the lower dry weights of the unstressed TL-3 compared to TL-2 and WT. Moreover, the soil-grown *PR10a*-overexpressing faba bean plants also displayed significantly improved drought tolerance in the greenhouse. Plant height and leaf number per plant of the *PR10a*-overexpressing plants were significantly higher than of the wide-type plants. Our results suggest that PR10a plants were able to cope with water deficit better than wild-type plants since the PR10a plants recovered after rewatering, whereas the wild-type plants failed. Thus, the ability to recover may be ascribed to the overexpression of *PR10a*, contributing to some physiological changes as seen in the gas exchange.

PR10 proteins play multiple roles in plant defense when exposed to abiotic and biotic stresses. Potato *PR10a* gene overexpression in transgenic faba bean lines resulted in greater drought and salt stress tolerance. Previous studies revealed that heterologous overexpression of *PR10* from several plant sources caused robust increases in plant tolerance against abiotic stresses. The overexpression of *SmPR10* from *Salix matsudana* Koidz in transgenic *Arabidopsis thaliana* enhanced plant resistance to NaCl stress [42]. Peanut *AhSIPR10* introduced into tobacco and banana conferred higher tolerance against salt and drought stresses, and the transgenic plants showed better photosynthetic efficiency under water stress [58,59]. Takeuchi et al. [60] reported evidence that overexpression of rice *RSOsPR10* resulted in tolerance to drought stress in rice and salt and drought stresses in bentgrass. Similarly, Hanafy et al. [15] reported the impact of *PR10a* from potato in transgenic faba bean that showed elevated tolerance to drought and salt stresses. Moreover, there is substantial evidence that PR10 proteins are also induced by other abiotic factors, such as cold, ultraviolet radiation, and oxidative stresses [31,34,36,38,44,61,62,63,64,65,66]. 

To evaluate whether *PR10a* overexpression in faba bean could improve gas-exchange parameters under abiotic stress, we estimated photosynthetic rate, electron transport rate, chlorophyll fluorescence, stomatal conductance, transpiration rate, and intercellular CO_2_ concentration in the wild-type and *PR10a*-overexpressing faba bean lines under abiotic stress. Under salt and drought stresses, significant decreases in gas-exchange parameters were recorded for the wild-type and transgenic lines compared to the normal growth conditions. However, transgenic lines showed significantly higher ϕPSII and photosynthetic rate attributes when compared with the wild-type under salt/drought stresses and rewatered conditions. Moreover, the transgenic faba bean plants showed a lower stomatal conductance and transpiration rate under drought stress and rewatered conditions. On the contrary, the transgenic lines showed a higher stomatal conductance, electron transport rate, transpiration rate, and intercellular CO_2_ attributes in transgenic lines when compared to wild-type plants under salt stress for 25 days. Abiotic stress limits photosynthetic capacity. Earlier studies reported that the reduction in plant growth is associated with a reduction in leaf chlorophyll fluorescence and photosynthesis [67,68]. The reduction in gas-exchange parameters due to salt stress could be interrelated to the damage of chlorophyll [69,70]. *PR10a*-overexpressing faba bean plants suffered less damage to their photosystem II because the plants had significantly higher ϕPSII and photosynthetic rate than wild-type, and this made it possible for the *PR10a*-overexpressing faba bean plants to maintain higher photosynthetic activity under abiotic stress. The obtained data showed that transgenic lines (especially transgenic line TL-3) had higher gas-exchange rates and had less change in fluorescence parameters. As a result, transgenic plants experienced less damage under stress and were more tolerant of salt and drought than wild-type plants. 

The SPAD value is an indicator of leaf chlorophyll content and allows inference about the functioning of the entire plant photosynthetic system [71]. Under high salinity, plant chlorophyll content generally decreased [72]. The main reason for the reduction in chlorophyll content caused by salinity is the blocking of electron transport [73]. From the obvious difference in chlorophyll (SPAD), it was clear that the transgenic faba bean plants were more greenish under salt stress than wild-type plants (Figure 6), which is a reliable indicator of protein synthesis, healthy growth and development of plants [74]. The obtained results are coherent with those obtained by [75], who noted a significant difference in chlorophyll content between salt-sensitive and tolerant sunflower lines under different NaCl salt concentrations. As a result, higher leaf chlorophyll content compared to those values in wild-type likely contributed to the enhanced stress tolerance of the transgenic faba bean plants. 

Osmotic adjustment is the main mechanism to conserve plant cell hydration under salt and drought stresses. The osmoprotectants accumulation realizes this prime cell tolerance response [6,8,15,76,77,78,79]. This process, in turn, helps the plant to retain its water balance and protects the cellular compartments [6,8,77]. Osmotic adjustment is widely used as an effective parameter for evaluating crop genotypes’ tolerance to osmotic stress [80]. Numerous studies had shown that salt stress induced a reduction in osmotic potential [6,76,77,81,82]. These reports also clearly showed that plants maintaining high leaf water content could tolerate salt stress. In the present study, the osmotic potential was estimated in the wild-type and the *PR10a*-overexpressing faba bean lines under salt stress (Table 3). Salinity resulted in a marked reduction in leaf osmotic potential in both wild-type and transgenic faba bean plants. However, this salt stress-induced reduction was significantly higher in wild-type as compared to that in transgenic lines. Moreover, the reduction in leaf osmotic potential in faba bean plants increases with the plant age. The reduction in leaf osmotic potential due to salt-stress has been widely reported in the literature [6,81,82,83]. The differences in leaf osmotic potential observed between wild-type and PR10a plants under salt stress could be ascribed to the changes in the ability of the plants to accumulate these osmolytes [84,85]. The remarkable reduction in leaf osmotic potential in wild-type depicts its inability to control the uptake of toxic ions, such as Na^+^. This suggests the enhanced salt stress tolerance of PR10a transgenic faba bean plants under salt stress as compared to wild-type.

It is well known that Na^+^ excess in the cytosol has a deleterious effect on the metabolism by disrupting intracellular K^+^ homeostasis, damaging membranes, inhibiting enzymes, and causing oxidation stress [80,86]. Previous reports have shown plant tolerance to salt stress is closely related to the maintenance of high cytosolic K^+^/Na^+^ homeostasis under stress [87,88,89]. For example, co-expression of ZxNHX and ZxVP1-1 in transgenic alfalfa plants resulted in a higher accumulation of Na^+^, K^+^, and Ca^2+^ in both leaves and roots [90]. Recently, [89] reported that K^+^/Na^+^ ratio, H_2_O_2_, proline content, malondialdehyde (MDA) content, ascorbate peroxidase (APX), and catalase (CAT) biochemical traits can be used for the identification of salt tolerance rice genotypes. In the present study and by analyzing the toxic effect of ions, it was found that transgenic faba bean overexpressing *PR10a* (TL-3) had low leaf Na^+^ and about equal levels of leaf K^+^ compared to wild-type plants, resulting in a higher K^+^/Na^+^ ratio (Table 4 and Figure S3). These results indicate that *PR10a* had a positive impact on the regulation of the salt tolerance of faba bean by regulating the K^+^/Na^+^ ratio to reduce the ion toxicity. 

One mechanism underlying salt stress tolerance is restricting Na^+^ and Cl^−^ uptake into functional tissues. The inhibition of the transportation of both ions from roots to leaves played an essential role in decreasing the toxicity of these ions in citrus [91]. These findings support the results of the current study. It was found that the faba bean wild-type plants showed significantly higher accumulation of Na^+^ in leaves than that in transgenic line TL-3 under salt stress regime (Table 4 and Figure S2). Interestingly, while the leaf Na^+^ content of TL-3 was lower than in wild-type plants, the Na^+^ was highly accumulated in the roots of this line compared to wild-type. These results suggest that TL-3 had a superior capacity for Na^+^ accumulation in roots, which counteracts salt stress in leaves by reducing leaf Na^+^ accumulation, possibly conferred by the high expression of the *PR10a* gene [15,44]. The obtained results are in line with those reported by [92], who observed that salt-sensitive faba bean cultivar accumulated higher concentrations of Cl^−^ and Na^+^ compared to tolerant cultivars, which had a negative effect on photosynthetic efficiency and reduced quantum efficiency of photosystem II. Similar observations were made in barley under salt stress [93]. Ebrahimi and Bhatla [94] reported a higher concentration of Na^+^ and Cl^−^ ions in sunflower grown under salt stress conditions. In addition, they described the growth reduction of sunflower due to Na^+^ and Cl^−^ toxicity. The results of the present study are in coherence with those reported by [29], who showed that PR10 transgenic tobacco plants accumulate a lower amount of Na^+^ ions than wild-type tobacco plants under 200 mM NaCl concentration. Similarly, Najar et al. [95] reported the toxic effect of Na^+^ and Cl^−^ in the leaf tissue of model legume plant *Medicago truncatula* L. which had a negative effect on water availability and cell turgor. The obtained results of the current study suggested that overexpression of the *PR10a* gene also had a role in the regulation of Na^+^ homeostasis under salt stress, by which PR10a transgenic faba bean plants conferred salt tolerance. The obtained data can be ascribed to various harmful effects of salinity on wild-type faba bean, including nutrient imbalance, osmotic effect, accumulation of toxic levels of Na^+^ and Cl^−^ in plant leaves, and reactive oxygen species (ROS)-induced oxidative stress. 

The results revealed that *PR10a* overexpression improves faba bean tolerance to drought and salt stresses by modulating gas-exchange attributes, regulating ions homeostasis, and improving photosynthesis under stress. Therefore, it can be concluded that the *PR10a* gene from potato could serve as a candidate for enhancing water stress tolerance in faba bean and likely other crops as well. Our results motivate us to carry out further research work to dissect the mode of action of the *PR10a* gene from potato in greater depth. Further characterization of PR10a faba bean plants at the biochemical and molecular level might provide an insight into the exact nature and role of the PR10a protein in stress tolerance. In particular, exploring the involvement of the PR10 protein in phytohormone signaling and the regulatory network of *PR10a* can provide additional information on the possible mode of action of the PR10 protein underlying abiotic stress tolerance. 

## 4. Materials and Methods 

### 4.1. Plant Materials and Growing Conditions

Transgenic faba bean (*Vicia faba* L. cv. Tattoo, a tannin-free cultivar) lines that contain a pathogenesis-related protein (PR10a) from potato under control of the mannopine synthetase (MAS) promoter, herbicide resistant gene (*bar*), and luciferase gene were obtained in our previous work [15]. Two independently transformed stable transgenic lines (TL-2 and TL-3) along with wild-type (WT) plants were grown under controlled greenhouse conditions at the Leibniz University Hannover, Germany. Seeds were germinated in rock-wool in a greenhouse under near-ideal conditions temperature regime (25/22 °C day/night) and a photoperiod of 16/8 h (light/dark). Twenty-five days after sowing, seedlings were transplanted onto styrofoam floating in a container filled with 25 L of nutrient solution. There were two plants per container. Each liter of the nutrient solution contained 0.5 g FERTY^®^ MEGA 2 (Planta GmbH, Regenstauf, Germany, 0.9 mM NO_3_^−^, 1.5 mM NH_4_^−^, 2.8 mM K^+^, 3.0 mM Ca^2+^, 0.4 mM Mg^2+^, 0.4 mM H_2_PO_4_, as well as adequate amounts of the micronutrients). The pH value was adjusted to 6.0–6.2 by 1% sulfuric acid. The photoperiod was adjusted to 14 h light and 10 h dark, and the temperature was maintained at 20–24 °C day/16–18 °C night, and relative humidity (65–70%), photosynthetic photon flux density (PPFD) was approximately 300 µmol m^−2^ s^−1^ and irrigation was with 500 mL as a constant volume of water for all plants every two days. The seeds of the fourth progeny of transgenic line TL-3 and three different progenies of transgenic line TL-2 (TL-2.2, TL-2.3, and TL-2.4) were used in the current study. Untransformed seeds and negative segregants from the transgenic lines were used as WT controls in these experiments. 

### 4.2. PCR Analysis of Transgenic Plants 

Total DNA was extracted from the young leaves of transgenic lines and WT plants (control) according to the method described previously [96] and subjected to standard PCR protocol using specific primers for the *PR10a* gene. Cycling conditions were: 94 °C, 5 min; (1 min at 94 °C, 1 min at 57 °C, 1 min at 72 °C) 30 cycles, then a final step of 10 min at 72 °C was included. The forward and reverse primers for the *PR10a* gene were PR10-For 5^’^-ATGGGTGTCACTAGCTATACACATG-3’ and PR10-Rev 5’-TTAAGCGTAGACAGAAG-GATTGGC-3’ amplifying a 480-bp *PR10a* sequence. 

### 4.3. Leaf Paint Test

The transgenic plants were tested in the greenhouse for the expression of the bar gene by painting the leaflets with BASTA^®^ (a commercial formulation of PPT containing 200 g/L ammonium glufosinate, Hoechst Ltd., Frankfurt, Germany) diluted to a concentration of 200–300 mg/L ammonium glufosinate. The opposite leaflet of each pair was marked and left untreated as a control.

### 4.4. Stress Treatments 

For drought stress treatments, three progenies of the transgenic line TL-2 (TL-2.2, TL-2.3, TL-2.4) and WT faba bean plants (20–30 plants of each line) were watered normally (every two days) to field capacity for six weeks, after which water was withdrawn for two weeks. The weights of the pots were measured every day to observe the gradual water decrease in the soil. 

For salt stress treatments, five days after transplanting under normal conditions, at least 4 plants from both WT and transgenic lines were exposed to different salinity conditions by adding NaCl to the hydroponic culture system to a level of 50 mM on day 0, to a level of 100 mM on day 3, and then to a level of 150 mM on day 10 for 19 days. Finally, after a total of 29 days of salt stress, a recovery period was given to the stressed plants by replenishing nutrient solution containing FERTY^®^ MEGA 2 without NaCl.

### 4.5. Phenotyping and Physiological Analysis of Transgenic Plants

Plant height was determined by measuring the length from the top of the shoot apex to the base of the stem. To determine biomasses, all plants were clipped and weighed to determine the fresh and dry weight data. Leaf number, branch number, root length were also measured. Gas exchange measurements, including quantum efficiency of photosystem II electron transport (ϕPSII), maximum efficiency of photosystem II photochemistry in the light (Fv’/Fm’), net photosynthetic rate (*A*_n_), stomatal conductance (*g*_s_), transpiration rate (*E*), and intercellular CO_2_ concentration (*C*_i_) were determined on light-adapted leaves using a portable gas exchange system (Li-6400XT, Li-Cor Inc., Lincoln, NE, USA) coupled with an integrated fluorescence chamber head (Li-6400-40 leaf chamber fluorometer; Li-Cor Inc., Lincoln, NE, USA). All measurements were performed between 09.00 h and 13.30 h. During measurements, leaf temperature, CO_2_ concentration, and light conditions were set at 25 °C, 400 µmol mol^−1^, and 1200 µmol m^−2^ s^−1^ PAR, respectively. In the drought stress experiment, measurements were taken on the third to seventh fully expanded leaves of the last fully emerged leaves on days 4, 10, 11, 14 after withholding water and two days after rewatering. Averages of all the measurements were calculated.

In the salt stress experiment, the leaf gas exchange measurements were conducted before salt stress application (day 0) and every third day from the salt application of 100 mM NaCl until the end of the stress application. Finally, the leaf gas exchange measurements were performed 5 days after lifting the salt stress. A portable chlorophyll meter (SPAD-502; Minolta Camera, Osaka, Japan) was used to measure leaf greenness on day 25 after salt stress application. Furthermore, leaf osmotic potential was measured 14 and 28 days after salt application using a vapor pressure psychrometer (Wescor HR-33 T with C-52 sample chamber, EliTech, Pureaux, France). After 29 days of salt stress followed by 14 days of the recovery phase (total 43 days), the plants were harvested, and the biomass of the plants as fresh/dry weights of leaf, stem, root, and total plant was measured. Finally, K^+^, Ca^2+^, Mg^2+^, Na^+^ ion contents in the plant materials were determined using an atomic absorption spectrometer, flame technique (Perkin Elmer 1100B, Flame: acetylene/air) according to (Kalra et al., 1998). Chloride ions (Cl^−^) content in the samples was determined using a potentiometric titration of chloride. 

### 4.6. Statistical Analyses 

The data collected were statistically analyzed by two-way-ANOVA. Upon significant interaction indicated by ANOVA, the means were compared within each treatment by Dunnett’s test using wild-type (WT) as control. Statistical analysis was done by packages ‘stats’ and ‘DescTools’ using the R studio program according to R Core Team [97]. The experiments were arranged in a completely randomized design with at least three replicates for each transgenic line and WT.

## Figures and Tables

**Table 1:** Fresh weights and branch number of transgenic faba bean lines TL-2, TL-3, and wild-type (WT) after 29 days of salt stress followed by a 14-day recovery period. Relative values of fresh weights under stress compared to the non-stressed condition are also shown. Values are the means ± SE. Asterisks indicate significant differences within treatment by Dunnett’s test using WT as control upon significant interaction indicated by two-way ANOVA. *** *p* < 0.001, ** *p* < 0.01, * *p* < 0.05, ns insignificance with *p* > 0.05.

Treatment	Line	Fresh Weight per Plant (g)				Branch Number per Plant
		Total	Leaf	Stem	Root	
Salt-stressed	TL-2	60.6 ± 12.6	23.7 ± 6.12	29.8 ± 6.60 ^ns^	7.07 ± 0.090	2.0 ± 0.0 ^ns^
	TL-3	107 ± 14.2	41.9 ± 5.08	50.0 ± 6.99 ^ns^	14.6 ± 2.27	3.75 ± 0.25 **
	WT	37.6 ± 10.2	15.3 ± 5.04	17.2 ± 4.76	5.10 ± 0.868	0.667 ± 0.333
Non-stressed	TL-2	349 ± 48.1	144 ± 15.3	150 ± 16.4 **	38.8 ± 10.0	8.67 ± 0.333 ^ns^
	TL-3	288 ± 38.9	103 ± 13.4	108 ± 5.66 ^ns^	77.5 ± 30.4	6.67 ± 0.882 ^ns^
	WT	239 ± 54.7	74.5 ± 38.4	75.0 ± 28.4	89.1 ± 12.1	7.5 ± 1.50
Two-way ANOVA	Treatment (T)	***	***	***	***	***
	Line (L)	*	**	**	ns	**
	T × L	ns	ns	*	ns	*
Relative value under stress	TL-2	0.174 ± 0.036 ^ns^	0.164 ± 0.043 ^ns^	0.199 ± 0.044 ^ns^	0.182 ± 0.002 *	
	TL-3	0.370 ± 0.049 *	0.408 ± 0.049^ns^	0.464 ± 0.065 ^ns^	0.189 ± 0.029 *	
	WT	0.158 ± 0.043	0.205 ± 0.068	0.230 ± 0.063	0.057 ± 0.010	

**Table 2:** Dry weights of transgenic faba bean lines TL-2, TL-3, and wild-type (WT) after 29 days of salt stress followed by a 14-day recovery period. Relative values of dry weights under stress compared to the non-stressed condition are also shown. Values are the means ± SE. Asterisks indicate significant differences within treatment by Dunnett’s test using WT as control upon significant interaction indicated by two-way ANOVA. *** *p* < 0.001, * *p* < 0.05, ns insignificance with *p* > 0.05.

Treatment	Line	Dry Weight per Plant (g)			
		Total	Leaf	Stem	Root
Salt-stressed	TL-2	9.92 ± 3.37	5.73 ± 1.50	3.55 ± 1.66	0.64 ± 0.21
	TL-3	10.8 ± 1.15	5.76 ± 0.557	4.21 ± 0.618	0.82 ± 0.083
	WT	6.37 ± 0.632	4.11 ± 0.248	1.88 ± 0.255	0.37 ± 0.151
Non-stressed	TL-2	34.3 ± 4.28	14.5 ± 1.86	17.6 ± 1.96	2.25 ± 0.553
	TL-3	25.8 ± 2.32	9.93 ± 1.07	12.0 ± 1.17	3.85 ± 1.25
	WT	32.6 ± 5.71	13.2 ± 1.83	14.8 ± 3.60	4.63 ± 0.28
Two-way ANOVA	Treatment (T)	***	***	***	***
	Line (L)	*	*	*	ns
	T × L	ns	ns	ns	ns
Relative value under stress	TL-2	0.289 ± 0.098 ^ns^	0.394 ± 0.104 ^ns^	0.202 ± 0.095 ^ns^	0.284 ± 0.093 *
	TL-3	0.418 ± 0.045 *	0.580 ± 0.056 *	0.351 ± 0.052 *	0.213 ± 0.022 ^ns^
	WT	0.195 ± 0.020	0.313 ± 0.019	0.127 ± 0.017	0.080 ± 0.033

**Table 3:** Leaf osmotic potentials of transgenic faba bean lines TL-2, TL-3, and wild-type (WT) after 14 and 28 days of salt stress. Values are means ± SE. Asterisks indicate significant differences within treatment by Dunnett’s test using WT as control upon significant interaction indicated by two-way ANOVA. *** *p* < 0.001, ** *p* < 0.01, * *p* < 0.05, ns, insignificance with *p* > 0.05.

Treatment	Line	Osmotic Potential (MPa)	
		14 Days	28 Days
Salt-stressed	TL-2	−1.01 ± 0.025 *	−1.37 ± 0.060 **
	TL-3	−1.18 ± 0.048 ^ns^	−1.31 ± 0.077 ***
	WT	−1.20 ± 0.051	−1.91 ± 0.249
Non-stressed	TL-2	−0.739 ± 0.048 ^ns^	−0.683 ± 0.044 ^ns^
	TL-3	−0.702 ± 0.050 ^ns^	−0.690 ± 0.054 ^ns^
	WT	−0.708 ± 0.078	−0.735 ± 0.050
Two-way ANOVA	Treatment (T)	***	***
	Line (L)	ns	**
	T × L	*	*

**Table 4:** Sodium content of transgenic faba bean lines TL-2, TL-3, and wild-type (WT) after 29 days of salt stress followed by a 14-day recovery period. Values are means ± SE. Asterisks indicate significant differences within treatment by Dunnett’s test using WT as control upon significant interaction indicated by two-way ANOVA. *** *p* < 0.001, ** *p* < 0.01, * *p* < 0.05, ns insignificance with *p* > 0.05.

Treatment	Line	Sodium Content (mg Na^+^ g^−1^ DW)		
		Leaf	Stem	Root
Salt-stressed	TL-2	35.4 ± 5.07 *	38.8 ± 8.95	12.1 ± 0.355 ^ns^
	TL-3	30.7 ± 1.98 **	17.5 ± 7.57	28.3 ± 4.37 **
	WT	55.1 ± 8.79	16.5 ± 12.8	11.6 ± 3.62
Non-stressed	TL-2	2.62 ± 0.150 ^ns^	20.9 ± 16.8	7.32 ± 0.11 ^ns^
	TL-3	2.56 ± 0.282 ^ns^	23.2 ± 10.2	8.66 ± 1.17 ^ns^
	WT	2.76 ± 0.03	17.2 ± 13.6	6.54 ± 1.40
Two-way ANOVA	Treatment (T)	***	ns	**
	Line (L)	**	ns	**
	T × L	*	ns	*p* = 0.0583

**Table 5:** Plant height and number of leaves per plant of different generations of transgenic faba bean line TL-2 and wild-type (WT) after 14 days of drought stress. Asterisks indicate significant difference within treatment by Dunnett’s test using WT as control upon significant interaction indicated by two-way ANOVA. *** *p* < 0.001, ** *p* < 0.01, ns insignificance with *p* > 0.05.

Treatment	Line	Plant Height (m)	Number of Leaves per Plant
Drought-stressed	TL-2.2	0.630 ± 0.010 ***	21.0 ± 3.0
	TL-2.3	0.630 ± 0.010 ***	20.7 ± 0.3
	TL-2.4	0.470 ± 0.010 ***	16.0 ± 2.0
	WT	0.340 ± 0.030	10.0 ± 2.0
Non-stressed	TL-2.2	0.645 ± 0.005 ***	21.5 ± 2.5
	TL-2.3	0.655 ± 0.005 ***	22.5 ± 2.5
	TL-2.4	0.500 ± 0.010 ^ns^	16.0 ± 2.0
	WT	0.505 ± 0.015	15.0 ± 1.0
Two-way ANOVA	Treatment (T)	***	ns
	Line (L)	***	**
	T × L	**	ns

**Table 6:** Net photosynthetic rate (*A*_n_), quantum efficiency of photosystem II electron transport under 1200 µmol m^−2^ s^−1^ PAR (ϕPSII), stomatal conductance (*g*_s_), transpiration rate (*E*), and intercellular CO_2_ concentration (*C*_i_) of different generations of transgenic faba bean line TL-2 after 14 days of drought stress or after 14 days of drought stress followed by a 2-d recovery period. Asterisks indicate significant differences within treatment by Dunnett’s test using wild-type (WT) faba bean as control upon significant interaction indicated by two-way ANOVA. *** *p* < 0.001, ** *p* < 0.01, * *p* < 0.05, ns insignificance with *p* > 0.05.

Treatment	Line	*A*_n_ (µmol CO_2_ m^−2^ s^−1^)	ϕPSII	*g*_s_ (mol H_2_O m^−2^ s^−1^)	*E* (mmol H_2_O m^−2^ s^−1^)	*C*_i_ (µmol CO_2_ mol^−1^)
Drought-stressed	TL-2.2	6.30 ± 0.431 ^ns^	0.169 ± 0.006 *	0.065 ± 0.020	1.13 ± 0.149	209 ± 67.4 **
	TL-2.3	8.04 ± 1.90 *	0.154 ± 0.006 ^ns^	0.073 ± 0.001	1.33 ± 0.048	208 ± 39.2 **
	TL-2.4	5.44 ± 0.647 ^ns^	0.169 ± 0.007 *	0.073 ± 0.023	1.25 ± 0.380	234 ± 52.1 *
	WT	3.02 ± 1.11	0.056 ± 0.010	0.095 ± 0.027	1.60 ± 0.346	340 ± 8.20
Drought-stressed + 2-d recovery	TL-2.2	9.42 ± 0.749 *	0.222 ± 0.031 *	0.113 ± 0.002	2.12 ± 0.332	249 ± 12.4 ^ns^
	TL-2.3	10.7 ± 0.377 **	0.184 ± 0.001 ^ns^	0.115 ± 0.004	2.21 ± 0.099	232 ± 5.24 *
	TL-2.4	8.50 ± 0.366 ^ns^	0.156 ± 0.001 ^ns^	0.103 ± 0.005	2.06 ± 0.047	250 ± 1.38 ^ns^
	WT	3.87 ± 0.145	0.084 ± 0.003	0.136 ± 0.023	2.43 ± 0.339	338 ± 9.89
Non-stressed	TL-2.2	9.17 ± 0.768 ^ns^	0.153 ± 0.018 ^ns^	0.116 ± 0.008	1.97 ± 0.062	255 ± 12.9 ^ns^
	TL-2.3	9.96 ± 0.844 ^ns^	0.138 ± 0.025 ^ns^	0.152 ± 0.024	2.50 ± 0.411	269 ± 9.38 ^ns^
	TL-2.4	9.21 ± 0.602 ^ns^	0.169 ± 0.013 ^ns^	0.130 ± 0.016	2.13 ± 0.181	265 ± 11.5 ^ns^
	WT	9.67 ± 1.26	0.197 ± 0.028	0.137 ± 0.028	2.28 ± 0.435	262 ± 15.1
Two-way ANOVA	Treatment (T)	***	ns	**	**	ns
	Line (L)	***	ns	ns	ns	**
	T × L	*	**	ns	ns	*

## Data Availability

Data is contained within the article or supplementary material.

## Supplementary Materials

The following are available online at https://www.mdpi.com/2223-7747/10/1/173/s1, Figure S1: Fresh weights and branch numbers data. Figure S2: Changes in the chloride (Cl^−^) contents. Figure S3: Changes in potassium (K^+^) contents. Figure S4: Changes in the calcium (Ca^2+^) contents. Figure S5: Changes in the magnesium (Mg^2+^) contents.
