# Supplementary material for: Enhanced Abiotic Stress Tolerance of Vicia faba L. Plants Heterologously Expressing the PR10a Gene from Potato
Source: Plants (Basel). 2021 Jan 18;10(1):173. doi: 10.3390/plants10010173 (PMC7831506; doi:10.3390/plants10010173)
Supplement: Supplementary file 1 [file plants-10-00173-s001.pptx]

## Slide 1
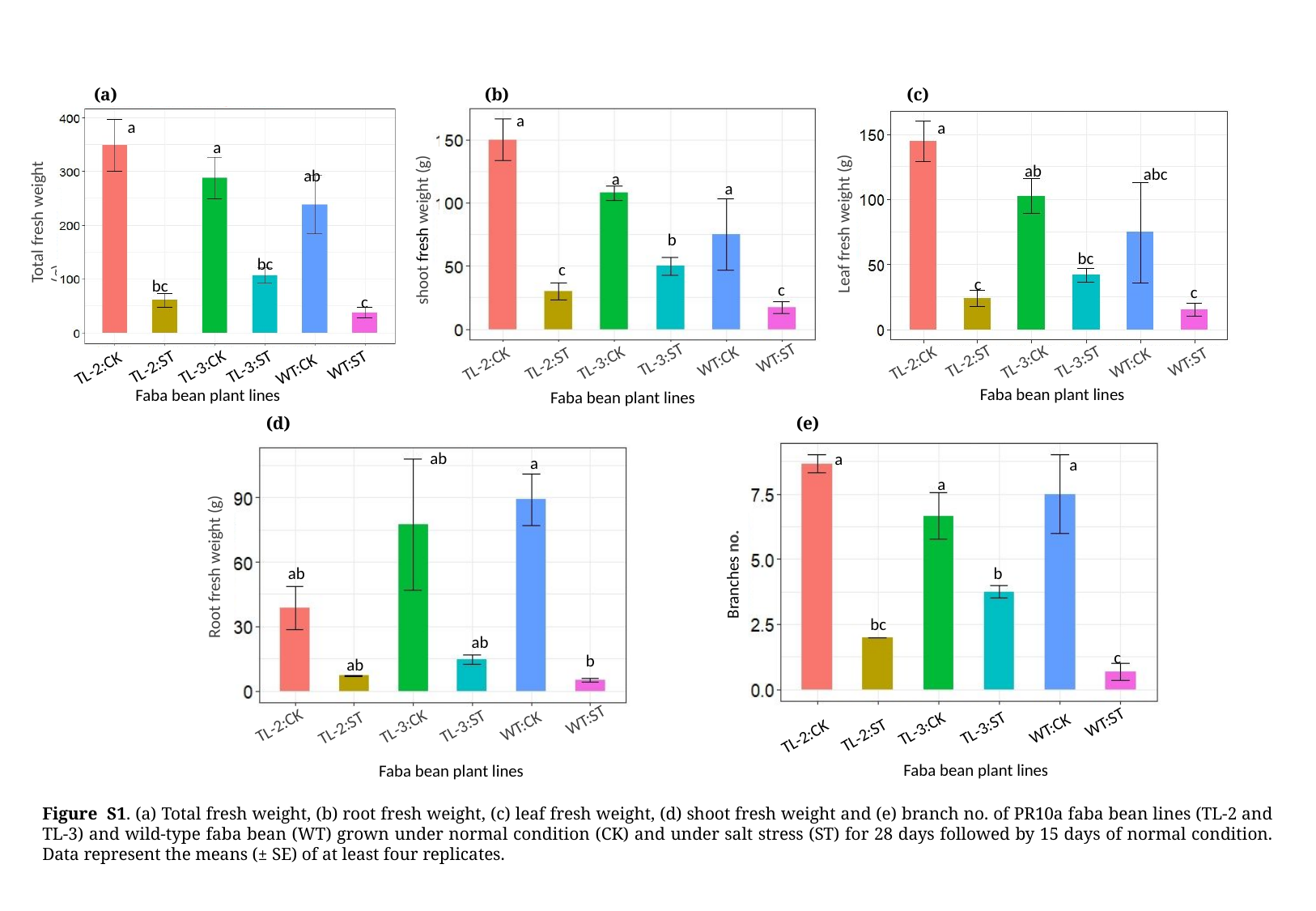

a
 ab
bc
 c
bc
TL-2:CK
WT:CK
WT:ST
TL-3:ST
TL-2:ST
TL-3:CK
 a
 a
Total fresh weight (g)
(a) 			 (b) 			 (c)
 a
ab
ab
abc
bc
bc
c
bc
c
c
Leaf fresh weight (g)
WT:CK
TL-2:CK
TL-2:ST
TL-3:ST
TL-3:CK
WT:ST
a
a
a
b
c
c
shoot fresh weight (g)
WT:CK
TL-2:CK
WT:ST
TL-3:ST
TL-3:CK
TL-2:ST
Faba bean plant lines
Faba bean plant lines
Faba bean plant lines
(d) 				 (e)
 a
a
a
a
 a
b
b
c
b
 bc
c
Branches no.
WT:CK
TL-2:CK
WT:ST
TL-3:ST
TL-3:CK
TL-2:ST
ab
a
ab
ab
b
ab
Root fresh weight (g)
TL-2:CK
WT:CK
WT:ST
TL-3:ST
TL-3:CK
TL-2:ST
Faba bean plant lines
Faba bean plant lines
Figure S1. (a) Total fresh weight, (b) root fresh weight, (c) leaf fresh weight, (d) shoot fresh weight and (e) branch no. of PR10a faba bean lines (TL-2 and TL-3) and wild-type faba bean (WT) grown under normal condition (CK) and under salt stress (ST) for 28 days followed by 15 days of normal condition. Data represent the means (± SE) of at least four replicates.

## Slide 2
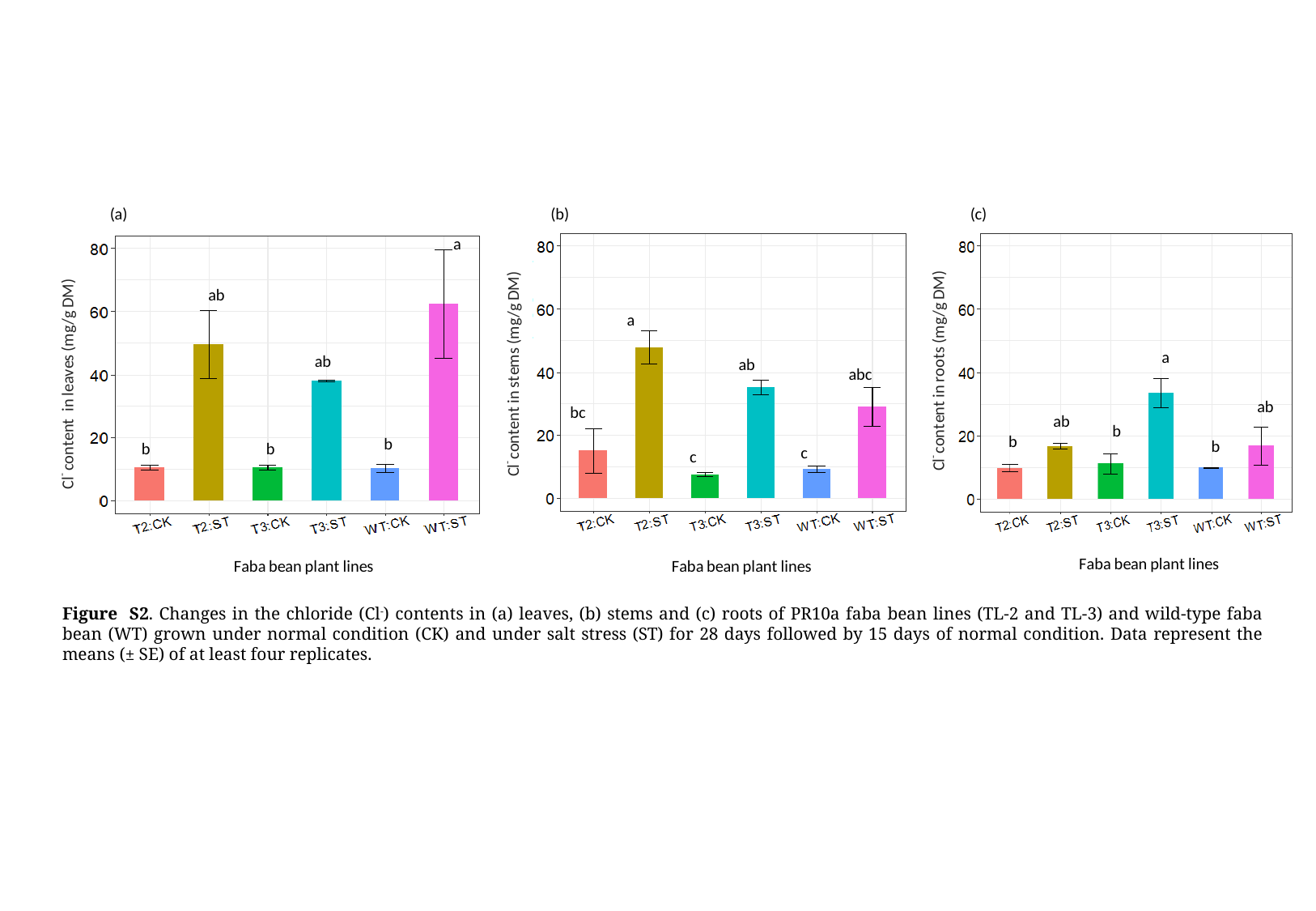

Figure S2. Changes in the chloride (Cl-) contents in (a) leaves, (b) stems and (c) roots of PR10a faba bean lines (TL-2 and TL-3) and wild-type faba bean (WT) grown under normal condition (CK) and under salt stress (ST) for 28 days followed by 15 days of normal condition. Data represent the means (± SE) of at least four replicates.

## Slide 3
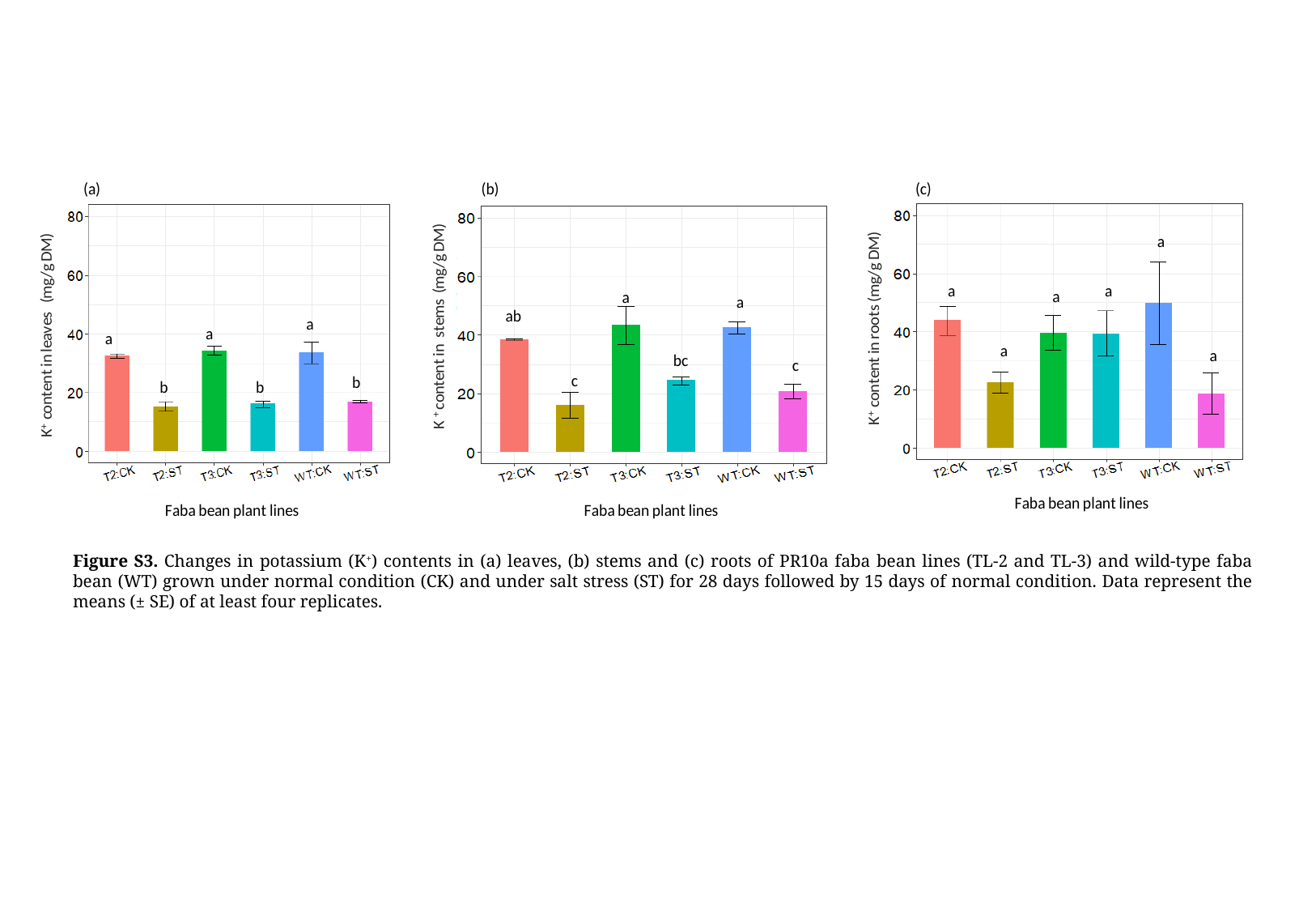

Figure S3. Changes in potassium (K+) contents in (a) leaves, (b) stems and (c) roots of PR10a faba bean lines (TL-2 and TL-3) and wild-type faba bean (WT) grown under normal condition (CK) and under salt stress (ST) for 28 days followed by 15 days of normal condition. Data represent the means (± SE) of at least four replicates.

## Slide 4
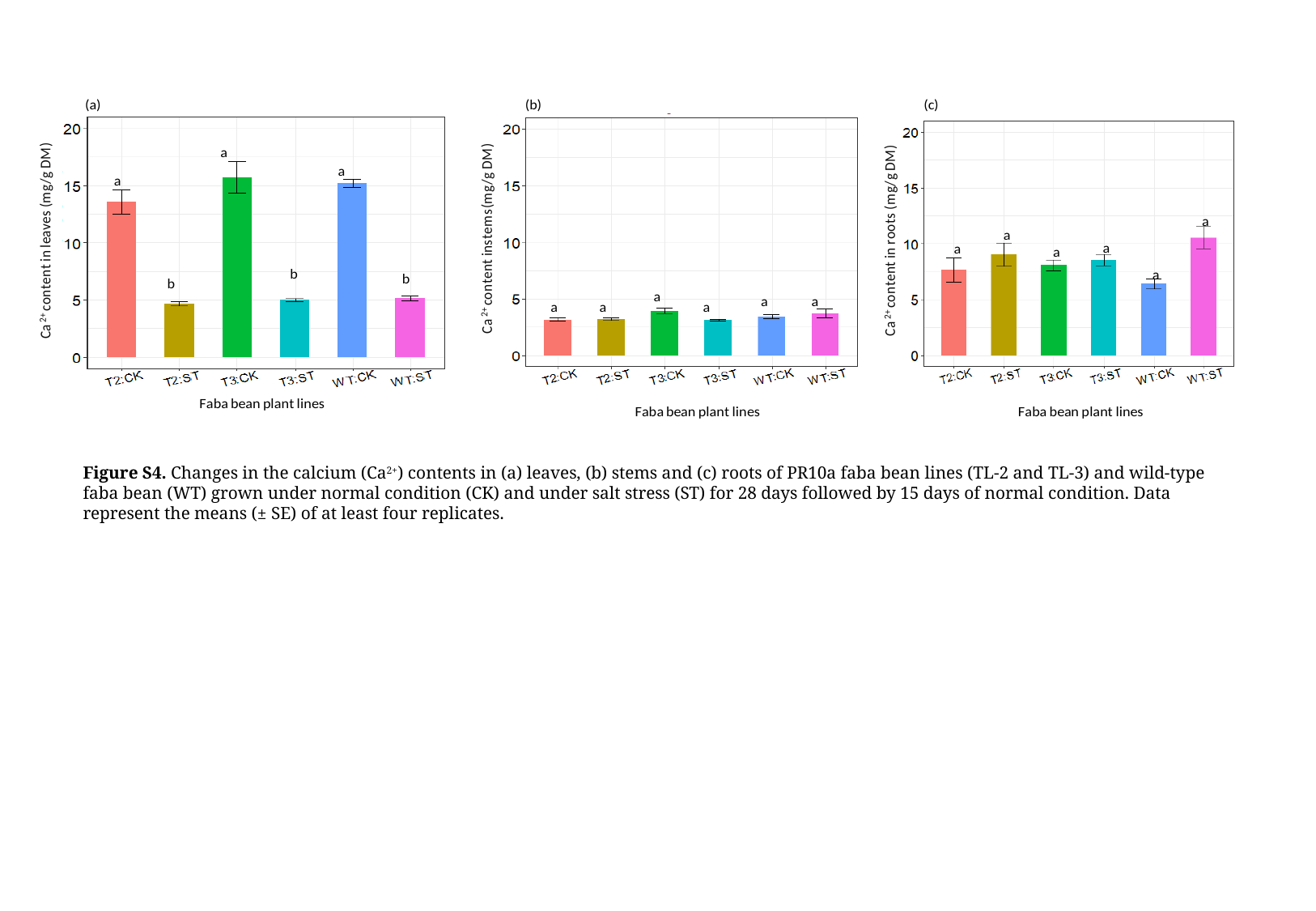

Figure S4. Changes in the calcium (Ca2+) contents in (a) leaves, (b) stems and (c) roots of PR10a faba bean lines (TL-2 and TL-3) and wild-type faba bean (WT) grown under normal condition (CK) and under salt stress (ST) for 28 days followed by 15 days of normal condition. Data represent the means (± SE) of at least four replicates.

## Slide 5
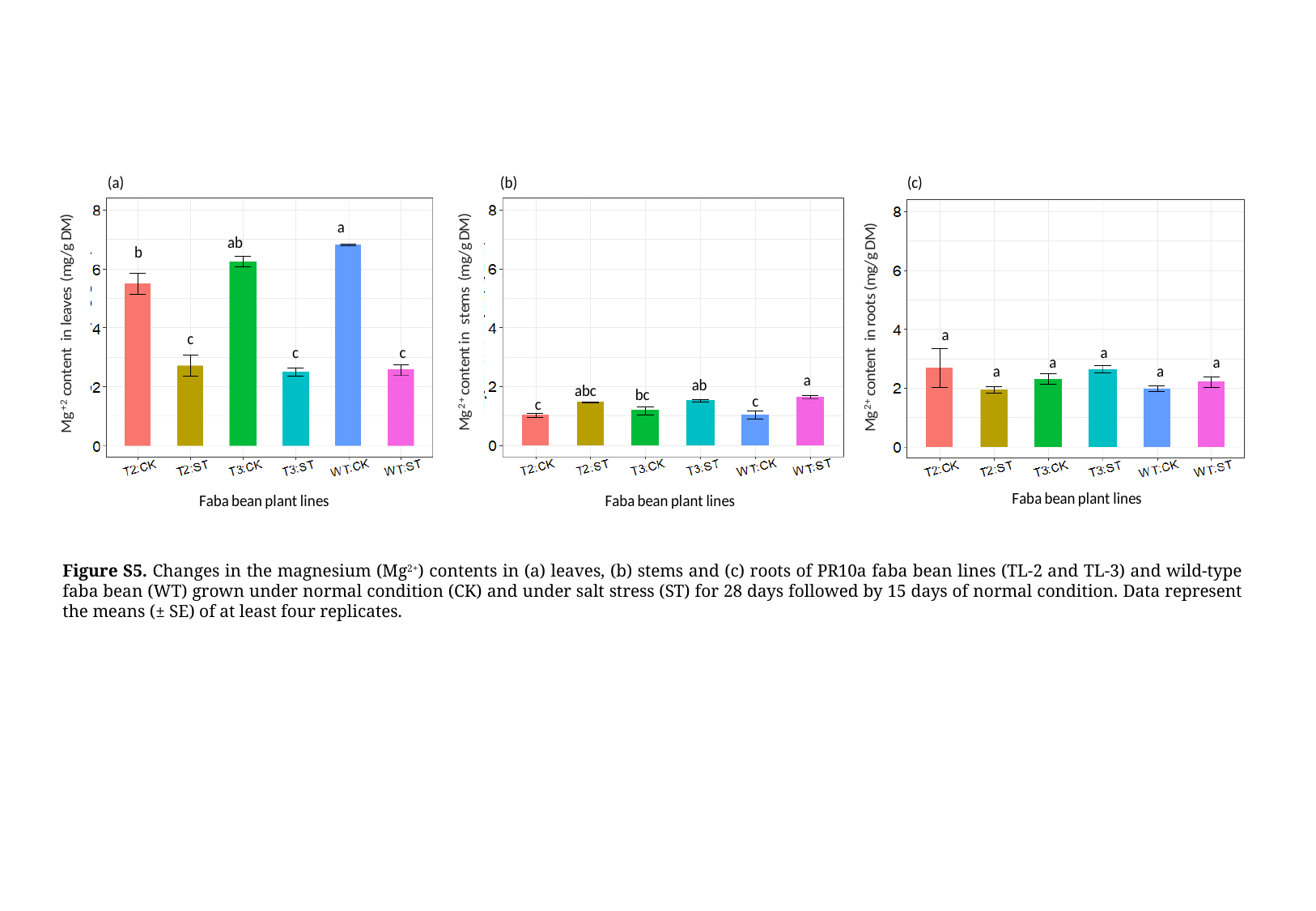

Figure S5. Changes in the magnesium (Mg2+) contents in (a) leaves, (b) stems and (c) roots of PR10a faba bean lines (TL-2 and TL-3) and wild-type faba bean (WT) grown under normal condition (CK) and under salt stress (ST) for 28 days followed by 15 days of normal condition. Data represent the means (± SE) of at least four replicates.
